# A Case-Control Study of Risk Factors for Bovine Brucellosis Seropositivity in Peninsular Malaysia

**DOI:** 10.1371/journal.pone.0108673

**Published:** 2014-09-29

**Authors:** Mukhtar Salihu Anka, Latiffah Hassan, Siti Khairani-Bejo, Mohamed Abidin Zainal, Ramlan bin Mohamad, Annas Salleh, Azri Adzhar

**Affiliations:** 1 Department of Veterinary Pathology and Microbiology, Faculty of Veterinary Medicine, Universiti Putra Malaysia, Serdang, Selangor, Malaysia; 2 Department of Agribusiness and information system, Faculty of Agriculture, Universiti Putra Malaysia, Serdang, Selangor, Malaysia; 3 Veterinary Research Institute, Ipoh Perak, Malaysia; 4 Epidemiology and Surveillance Unit, Department of Veterinary Services, Putrajaya, Malaysia; University of Brighton, United Kingdom

## Abstract

Bovine brucellosis was first reported in Peninsular Malaysia in 1950. A subsequent survey conducted in the country revealed that the disease was widespread. Current knowledge on the potential risk factors for brucellosis occurrence on cattle farms in Malaysia is lacking. Therefore, we conducted a case-control study to identify the potential herd-level risk factors for bovine brucellosis occurrence in four states in the country, namely Kelantan, Pahang, Selangor and Negeri Sembilan. Thirty-five cases and 36 controls of herds were selected where data on farm management, biosecurity, medical history and public health were collected. Multivariable logistic regression identified that *Brucella* seropositive herds were more likely to; have some interaction with wildlife (OR 8.9, 95% CI = 1.59–50.05); originated from farms where multiple species such as buffalo/others (OR 41.8, 95% CI = 3.94–443.19) and goat/sheep (OR 8.9, 95%Cl = 1.10–71.83) were reared, practice extensive production system (OR 13.6, 95% CI 1.31–140.24) and have had episodes of abortion in the past (OR 51.8, 95% CI = 4.54–590.90) when compared to seronegative herds. Considering the lack of information on the epidemiology of bovine brucellosis in peninsular Malaysia and absence of information on preventing the inception or spread of the disease, this report could contribute to the on-going area-wise national brucellosis eradication program.

## Introduction

Bovine brucellosis is a widespread livestock disease with worldwide distribution [1] caused by Gram-negative bacteria of the genus *Brucella*. [2]. In cattle, brucellosis is usually caused by *B. abortus*, but has also been attributed to *B. melitensis* and infrequently to *B. suis* [3], [4]. The disease is characterised by infertility, abortion among females, and orchitis and epididymitis in males [5]. Brucellosis causes serious economic losses to farmers and the government through direct production losses as well as additional costs for control and eradication programs [6]. Although bovine brucellosis has been controlled and eradicated in most of the developed nations, it remains a significant problem for both cattle and human health, especially in developing countries [7], [8].

As in most Southeast Asia countries, bovine brucellosis has been a problem among livestock for many years in Malaysia [9], [10]. The first evidence of the disease was reported at *Institut Haiwan* in Malaysia among imported cattle in 1950 [11], but it was effectively controlled through an intensive testing and slaughter program, accompanied by vaccination of young animals [12]. Sporadic cases of brucellosis continue to occur among local animals and, recently, Malaysian veterinary authorities observed an increasing trend in the seroprevalence of brucellosis among livestock [5], [13].

Studies of limited geographic localities to detect bovine brucellosis in Peninsular Malaysia have been carried out [9], [12], [14]; however, so far none have attempted to identify the risk factors for *Brucella* seropositivity among cattle. Knowledge about important determinants for *Brucella* seropositivity is vital, as these factors can be further explored in strategizing evidence-based disease control measures in the country. In this study, we assessed the role of several putative factors in the occurrence of bovine brucellosis among herds in Peninsular Malaysia and suggest how these factors can be modified to reduce the risk of the infection.

## Materials and Methods

### Ethics Statement

The study was approved by the Department of Veterinary Services, Putrajaya, Ministry of Agriculture Malaysia. A written consent was sought from every study participant before administering the questionnaire.

### Study area and study population

Malaysia is a Southeast Asian country located between 2.3167° North and 111.5500° East. It comprises West Malaysia (Peninsular Malaysia) and East Malaysia (Sabah and Sarawak on Borneo Island) separated by the South China Sea. The study was conducted in Peninsular Malaysia, which is comprised of 11 states and two federal territories and covers an area of 131,598 square kilometres bordering Thailand in the north and Singapore in the south. The country has a tropical climate with warm weather all year round. Rainfall varies throughout the year with an average of 2,400 mm (http://www.met.gov.my). Malaysia has a relatively small cattle population, with an estimated total cattle population in 2010 of 836,910 head [15]. Two main cattle breeds are encountered in Malaysia: Kedah-Kelantan, constituting a high percentage of the total beef cattle, and Local Indian Dairy (LID) cattle, which is the main dairy cattle population. Other breeds are also imported to increase production, including Brahman, Hereford, Aberdeen Angus, Droughtmaster, Bali, among others [16].

For this study we chose four states (Kelantan, Pahang, Selangor, Negeri Sembilan) based on the seroprevalence rates of *B. abortus* in each state in the years prior to the study. The veterinary authorities of Malaysia have been performing systematic and nationwide brucellosis testing and culling as part of the effort to control the infection among livestock in the country. At the time of the study, the estimated cattle population was 128,907 cattle in Kelantan, 47,227 in Negeri Sembilan, 169,312 in Pahang and 31, 236 in Selangor from 2,840, 1,212, 4,111 and 1,258 registered livestock premises, respectively [17].

### Study design

We performed a case-control study to determine the herd-level risk factors for bovine brucellosis between August 2011 and March 2012. Sampling was performed by selecting states with high seroprevalence of brucellosis based on the nationwide brucellosis serosurveillance data. Within each state, farms were identified via database of the serosurveillance program carried out by the veterinary authority of Malaysia in 2010. The list of herds in dataset of the year 2010 was used as the sampling frame for the herd selection. Stratified sampling within each state was performed to obtain seronegative and seropositive herds. We defined ‘case’ herds as those cattle herds which, within the previous one year, were found to have at least one seropositive confirmed by both the Rose Bengal Plate Test (RBPT) and the Complement Fixation Test (CFT) as prescribed by the OIE protocol at the Veterinary Research Institute (VRI), while ‘control’ herds were herds that had no seropositives. The CFT test have a reported sensitivity and specificity of 95% and 100% respectively [18]. From the list of seropositive and seronegative farms in each state, selection of farms were made randomly and lists of the selected farms were delivered to their respective state DVS for their approval, in addition to letters dispatched to individual farms. At the time of our study, some farms were no longer operational, and replacements for them were based on the recommendations of officials from the state DVS.

Sample size was calculated using OpenEpi software version 2.3 (OpenEpi, Atlanta, GA, USA) for an unmatched case-control study with α = 0.05, power = 80, ratio of cases to controls = 1.0, hypothetical proportion of exposure among controls = 30 and hypothetical proportion of exposure among cases = 65. A total of at least 31 herds each for the cases and control groups were required based on these assumptions. We selected a total of 71 herds (cases n = 35, controls n = 36) from different states; 20 herds from Kelantan (cases n = 10, controls n = 10), 16 from Negeri Sembilan (cases n = 8, controls n = 8), 18 from Selangor (cases n = 8, controls n = 10) and 17 from Pahang (cases n = 9, controls n = 8).

### Data collection

At each farm visit, state district veterinary officers accompanied the researchers to locate the farms and interview the farmers. The farmers were interviewed using a structured closed-ended questionnaire. The questionnaire sought information on farm demography, farm size, the size of the cattle population in the herd, breed of cattle, origin of the cattle, the system of farm management, biosecurity, medical history of the farm and the health aspect of the farmers.

### Data analysis

Descriptive statistics were generated for the study farms/herds in relation to the system of management, farm size, and origin of the cattle. A univariable logistic regression analysis examined the association between case-control status and potential risk factors. Variables were grouped into different categories of general farm characteristics, farm management, biosecurity, medical history of the farm and health aspect of the owners and their staff or family members. The strength of associations between case-control status and potential risk factors was analysed using odds ratio (OR) and 95% confidence interval (CI). Variables significant in the univariable analysis were tested for collinearity using the chi-square test for independence. A multivariable logistic regression was then constructed using a backward unconditional method to identify potential risk factors for bovine brucellosis; interactions were also tested for explanatory variables. Those explanatory variables significantly associated with case/control status in the univariable analysis (p≤0.05) were fitted into the multivariable analysis. Herd size was categorised into <15, 15–30 and>30 head of cattle; the age range of the cattle was categorized into <3, 3–6 and>6 years. The overall goodness of fit was accessed using the Hosmer-Lemeshow test. Information from the questionnaire was entered into a Microsoft excel spreadsheet (Microsoft Corporation) and data was imported into the SPSS version 20 software for statistical analysis (SPSS Inc. Chicago USA).

## Results

### Description of the study herds

The studied areas contained three management systems: free grazing (extensive system n = 34), feedlot (intensive system n = 19), and semi-intensive (n = 18). Malaysia has a large area cultivated with major crops such as oil palm, rubber, coconut etc. These plantations are integrated with livestock. The government introduced the integration system to improve livestock production and support farmers to eradicate poverty [19]. At the time of the study, Peninsular Malaysia has an estimated cattle population of 778,189 and most of these animals are raised under an integrated system [19], [20]. About 47.9% (34) of the farms we studied were extensive/integrated farms, 21 being cases and 13 being controls. Semi-intensive farms comprised 25.4%, with 10 cases and 8 controls, while intensive farms accounted for only 26.8%, comprising 4 cases and 15 controls. Out of the 71 herds in the study, 83.1% (59) herds were beef cattle, while 14.1% (10) were dairy herds and 2.8% (2) practiced both.

Cattle from 64.8% (46) of the herds sampled were from Malaysia, 19% (14) were imported from Australia, 8.5% (6) were from Thailand and the remaining 7% (5) were from other countries. The largest herds were in Kelantan with 800 (112±200) head of cattle, followed by Pahang with 517 (168±142) head, Selangor with 420 (132±112) head and Negeri Sembilan with 270 (127±121) head of cattle. About 94% (67) (34 cases and 33 controls) of farms used an open-housing system while 5.6% (4) (1 case and 3 controls) had closed housing systems. About 53.5% (38) of the farms sourced drinking water from a river while 28.2% (20) sourced water from the tap. A minority of the farmers 18.3% (13) indicated sourcing water from a pond. Of the studied herds, 90.1% (64) reported doing in-house breeding, while 9.9% (7) indicated doing breeding outside the farm. In response to whether other species of animal were kept on the farm, most of the interviewed farmers reported rearing other species on the farm: 54.9% (39) also rear goats, 10.3% (4) sheep, and 30.8% (13) buffalo, horses or deer.

Among the sampled farms, 49.3% (35) reported cleaning the farm every day while others cleaned less regularly and only 6 (8.5%) reported using disinfectant. About 54.9% of the farmers did not allow visitors into their farm. Well over half (88.7%) of those surveyed have no biosecurity facility on their farm. The majority of the farmers (51, or 71.8%) have no personal protective equipment (PPE) such as gloves, facemask and boots on the farm while only 28.2% (20) use PPE. Most of the farmers (50, or 70.4%) noted the presence of wildlife such as wild boars and tigers around their farms. Of the 71 herds participating in this study, 35.2% (25) possessed no isolation facilities while 64.8% (46) did. About 54.9% (39) of sampled herds had had abortion episodes previously. Only 19.7% (14) of the farmers reported culling seropositive animals while 46.5% (33) indicated treating these animals with unspecified drugs and 46.5% reported selling them.

The result from the univariable logistic regression analysis revealed that the production system, rearing multiple species of animals, the presence of wild life and history of abortion all have significant impact on the bovine brucellosis sero-status of cattle herds in Peninsular Malaysia (Table 1).

**Table 1:** Univariable analysis of potential risk factors for bovine brucellosis herd seropositivity in Peninsular Malaysia.

Variable	Category	Cases (n = 35)	Controls (n = 36)	eOR and 95% CI	P-value
**Management**					
System of production	Intensive	4	15	Ref	Ref
	Semi-intensive	10	8	4.7, 1.11–19.83	0.036
	Extensive	21	13	6.1, 1.65–22.27	0.007
State	Pahang	9	8	Ref	Ref
	N. Sembilan	8	8	0.9, 0.23–3.49	0.866
	Kelantan	10	10	0.9, 0.24–3.24	0.858
	Selangor	8	10	0.7, 0.19–2.69	0.616
Breed	Kedah-Kelantan	7	10	Ref	Ref
	Brahman	3	3	1.4, 0.22–9.26	0.708
	Local Indian Dairy	2	5	0.6, 0.09–3.83	0.564
	Kedah-Kelantan cross	6	3	2.9, 0.53–15.47	0.223
	Mixed	17	15	1.6, 0.49–5.32	0.427
Type	Dairy	2	8	Ref	Ref
	Beef	32	27	4.7,0.93–24.24	0.06
	Both	1	1	4.0,0.17–95.76	0.39
Age range of cattle	<3	9	8	Ref	Ref
	3–6	22	21	0.9, 0.30–2.87	0.901
	>6	4	7	0.5, 0.11–2.40	0.393
Herd size	<15	5	5	Ref	Ref
	15–30	2	7	0.3, 0.04–2.11	0.220
	>30	29	23	1.2, 0.30–4.52	0.823
Origin of cattle	Malaysia	21	25	Ref	Ref
	Imported	4	4	1.2, 0.27–5.35	0.820
	Both	10	7	1.7, 0.55–5.25	0.356
Housing	Close-house	1	3	Ref	Ref
	Open-house	34	33	0.3, 0.03–3.27	0.339
Water source	Tap	10	10	Ref	Ref
	Pond	4	9	0.4, 0.10–1.93	0.279
	River	21	17	1.2, 0.42–3.16	0.702
Breeding	In the farm	33	31	Ref	Ref
	Outside the farm	2	5	0.4, 0.07–2.08	0.262
Other species in the farm	No other species	21	11	Ref	Ref
	Buffaloes/Others	13	4	3.1, 1.15–8.09	0.007
	Goat/Sheep	11	11	1.9, 0.63–5.79	0.253
**Biosecurity**					
How often you clean farm	Daily	7	4	Ref	Ref
	Fortnightly	2	2	2, 0.23–17.33	0.529
	Monthly	1	1	2, 0.11–36.95	0.641
	Weekly	3	3	2.8, 0.90–8.37	0.075
	No cleaning	22	16	2, 0.318–12.59	0.460
Use of disinfectant	No	33	3	Ref	Ref
	Yes	32	3	1, 0.19–5.49	0.971
Visitors	No	16	23	Ref	Ref
	Yes	19	13	2.1, 0.81–5.44	0.126
Washing facilities	No	30	33	Ref	Ref
	Yes	5	3	1.8, 0.40–8.34	0.433
PPE	No	24	27	Ref	Ref
	Yes	11	9	1.4, 0.49–3.88	0.548
Wildlife	No	5	16	Ref	Ref
	Yes	30	20	4.8, 1.54–15.19	0.008
Isolation Facilities	No	14	11	Ref	Ref
	Yes	21	25	0.7, 0.25–1.76	0.406
**Medical History**					
History of abortion	No	8	24	Ref	Ref
	Yes	27	12	6.8, 2.36–19.29	0.001
Handling abortion	Cull	8	6	Ref	Ref
	Treat	20	13	1.2, 0.33–4.10	0.825
	No response	7	17	0.3, 0.08–1.22	0.094
Clinical sign*	No	2	6	Ref	Ref
	Yes	33	30	3.3, 0.62–17.60	0.160

eOR, exposure odds ratio; Ref, reference categories; CI, confidence interval, PPE, personal protective equipment,

*animal showing at least one of the clinical signs (orchitis, retained placenta, mastitis, weak foetus, decreased milk production, low conception rate).

### Multivariable logistic regression

The multivariable logistic regression results (Table 2) showed the association of various potential risk factors to herd-level seropositivity. The final model indicated that compared to seronegative herds, seropositive herds were significantly more likely to: have some level of interaction with wildlife (OR 8.9, 95% CI = 1.59–50.05), contained mix species of animals such as buffalo, horses, deer or dogs (OR 41.8, 95% CI = 3.94–443.19) and goats/sheep (OR 8.9, 95%Cl = 1.10–71.86), practice extensive farming system (OR13.6, 95% CI = 1.31–140.24) and have had history of abortion (OR 51.8, 95% CI = 4.54–590.90).

**Table 2:** Multivariable logistic regression of potential risk factors for bovine brucellosis herd seropositivity in Peninsular Malaysia.

Variable	Category	Cases (n = 35)	Controls (n = 36)	eOR and 95% CI	P-value
System of production	Intensive	4	15	Ref	Ref
	Semi-intensive	10	8	7.3, 0.88–60.82	0.065
	Extensive	21	13	13.6, 1.31–140.24	0.029
Other species on the farm	No other animals	21	11	Ref	Ref
	Buffalo/others	13	4	41.8, 3.94–443.19	0.002
	Goat/Sheep	11	11	8.9, 1.10–71.83	0.040
History of abortion	No	8	24	Ref	Ref
	Yes	27	12	51.8, 4.54–590.90	0.001
Wildlife	No	5	16	Ref	Ref
	Yes	30	20	8.9, 1.59–50.05	0.013

eOR, exposure odds ratio; Ref, reference categories; CI, confidence interval.

The Hosmer-Lemeshow goodness of fit test showed that the model fit the data well (X2 = 1.960, d.f. 8, p = 0.982). The chi-square test for independence showed no important collinearities between the predictive variables.

### Occupational risk and awareness among farmers about brucellosis

About 78% (56) of the farmers participating in the study reported that they had assisted the parturition process of cows on their farm and 71.8% (51) did not use basic PPE such as gloves or boots, especially when cleaning the farms. About 8.5% (6) (3 cases, 3 controls) reported consuming unpasteurized milk from their animals and 19.7% (14) have had episodes of fever, with one of the farmer experiencing undulant fever who was later diagnosed with brucellosis (Table 3)

**Table 3:** Potential risk of *Brucella* infection among cattle farmers.

Variable	Category	Number of farms (%) (n = 71)	Case farms (%) (n = 35)	Control farms (%) (n = 36)
Assist in delivery		56 (78.9)	33(58.9)	23(41.1)
Milk was consumed	Raw	2 (2.8)	1(50)	1(50)
	Boiled	5 (7.0)	2(40)	3(60)
	Never	64 (90.1)	32(50)	32(50)
Symptoms reported by farmers	Fever	14 (19.7)	8(57.1)	6(42.9)
	Undulant fever	1 (1.4)	1(100)	0(0)
	No	56 (78.9)	26(46.4)	30(53.6)
Diagnosed with brucellosis		1 (1.4)	1 (100)	0(0)

## Discussion

Several factors have been reported to be associated with bovine brucellosis [4], [21]–[24] in other parts of the world, such as the level of hygiene on the farm, the herd size, age of the cattle, sex, system of production, the presence of wildlife, and multiple livestock species within the herd. In this study, we found a significant association between the cattle production system and *Brucella* seropositivity, where cattle in an extensive system were found to be 13 times more likely to be exposed to *Brucella* infection compared to cattle in an intensive system. The observed increased risk of *Brucella* herd seropositivity based on the system of production confirms earlier findings by several authors [6], [23]. In their study, one group found that extensive production systems increased the risk of brucellosis by about 10.6-fold compared to cattle raised in an intensive system. In Malaysia, most cattle are raised in integrated farming systems that combine animal and crop farming simultaneously to enable synergistic interaction and greater overall output in terms of high productivity, profitability, sustainability, environmental safety, recycling, income round the year, adoption of new technology, energy savings, meeting fodder crises and generating more employment [25]–[27]. The extensive farms in this study practice integrated farming, and in this study we observed poor biosecurity and control of animal movements whereby the majority of the farms in this category had no fence or demarcation. Animals from various herds belonging to different owners freely interact and, in many instances, multiple cattle herds belonging to separate owners can be found on the same premises. This combination of a lack of biosecurity within the herd and poor control of animal movements plays an important role in the epidemiology of many diseases. In the case of brucellosis, an extremely contagious disease, the infection may be easily passed between animals following an abortion episode via pasture or feed contaminated with the organism, inhalation, conjunctiva inoculation, skin contamination, or from contaminated utensils used on infected colostrum for new born calves. Unplanned breeding, which is common in this type of production system, may also occur and sexual transmission plays a major role in the transmission of the disease [28], [29]. Most of the respondents (34, 47.9%) from our study confirmed that their animals mingled with other neighbouring cattle herds, and we believe that this maximizes contact between animals and facilitates the spread of the disease between infected and susceptible herds.

Another possible reason for the increased risk of exposure to *Brucella* organisms in the extensive farming system is contact with wildlife. Wild ungulates such as deer, elk and bison have been reported to be infected with *B. abortus* [30] and may serve as reservoirs of the organism transmitting the infection to susceptible cattle. Wild boars have also been incriminated in *B. abortus* infection [31]. We found that farms reporting sightings of wildlife had a 5.5-fold increased risk of *Brucella* seropositivity compared to farms that did not. Our finding is consistent with previous studies reporting the presence of *Brucella* antibodies in wildlife such as wild boars. The observed increased risk within herds that were exposed or interacted with wildlife could be a result of the high percentage of herds from extensive production systems in our study (47.9%), where cattle are allowed to move freely around the plantations and wild boars are also commonly seen roaming the plantations. Wild boars have been established as a reservoir of several infectious diseases [32], [33] including brucellosis [34]. In boars, brucellosis is usually caused by *B. suis* biovar 2 [34], [35]; however, *B. abortus* has also been isolated [36], [37]. In a limited sample size study by Sohayati et al. (2012), the seroprevalence of brucellosis in local wild boars was estimated at 62.5% (n = 8) [38]. The preferred host for *B. abortus* is cattle and other bovidae [39]; however, evidence of cross-infection across species has been reported by Donald (1990) [40] where *B. suis* was isolated from a cow. The presence of wildlife in areas where brucellosis is endemic among livestock is a concern as the wildlife may become infected as a result of spillover from infected cattle and become sustained in the wildlife population [34]. Infected wildlife may then serve as a source of *Brucella* infection during wildlife-livestock interactions.

The mixing of different species of animals, especially goats, buffalo and sheep with cattle is an important determinant for *Brucella* transmission in this study and has been reported elsewhere [41]–[43]. Our result shows that farms with buffalo, deer and horses were 24 times more likely to harbour cattle infected with the *Brucella* organism as compared to farms with only cattle. Similarly, farms with goats/sheep were 5 times more likely to harbour *Brucella* seropositive cattle compared to farms with only cattle. Cross-species infection with other *Brucella* species, especially *B. melitensis*, has been documented in cattle [43], [44]. Moreover, other non-cattle ungulates (such as buffalo, deer, and horses), feral swine and dogs may increase the risk of exposure to cattle because the bacteria have been isolated from each of these species [32], [45], [46] and animals such as buffalo may serve as maintenance hosts for the organism [47] [48]. Animals such as dogs have high mobility and may also serve as carriers of the organism [46], [49]. Under experimental conditions dogs can be infected with *B. abortus*, shed bacteria in reproductive discharges, and infect cattle [31].

In countries where extensive farming, especially combined with integrated farming, is widely practiced, the observation of clinical signs such as abortion and stillbirth is more complicated and can prove difficult. In Malaysia, brucellosis in animals has been marked by its indiscernible or unremarkable clinical symptoms, i.e. symptoms such as abortion storm have never been reported. This is probably a result of the unplanned breeding that is commonly practiced in animal production in developing countries [42]. In our study, even though abortion storm has not been reported, sporadic abortion is a significant determinant of bovine brucellosis seropositivity and proves that brucellosis must be included as a differential whenever abortion (even though in low numbers) was observed in a farm. This finding agrees with other studies [50], [51]. A study in India found the seroprevalence of brucellosis to be significantly higher in animals with a history of abortion than in those without while a study in Uganda also reported a history of abortion at the herd level to be a significant factor for brucellosis seropositivity. The primary source of the *Brucella* organism in the epidemiology of brucellosis in cattle is the uterine fluid and placenta or aborted foetus expelled by infected cattle during abortion or parturition [52]. Under optimum conditions, the *Brucella* organism can remain for 66 days in moist soil and up to 185 days in cold soil [51], [52]. Although not all abortions are due to brucellosis, abortion is a major clinical sign of the disease in cattle and therefore should be suspected whenever observed [30].

Brucellosis has major public health implications [53], with the disease mainly affecting people who work with livestock or animal products [54]. The findings from our study show that more than 50% of farmers assisted cows during parturition without using the most basic personal protective gear. Studies have shown that assisting animals during parturition increase the risk for brucellosis transmission to humans [55]. Indeed, the findings emphasized the need for basic education and knowledge of brucellosis transmission among farmers. Moreover, although the number of farmers consuming unpasteurised milk is insignificant, those who do have an increased risk for brucellosis. Therefore public health education and promotion on the prevention and control of common zoonotic diseases is certainly necessary for these populations.

Our study should be interpreted with caution because of several limitations. Its major limitation is selection bias, as some of the herds pre-selected were no longer operational upon visiting them. We attempted to reduce this bias by selecting another herd within the same district that had the same *Brucella* status using the sampling frame from the previous serosurveillance conducted by the DVS. Another potential bias is recall bias or selective recall by farmers when they were asked to recall events that may have occurred a few years prior to the study. Selective memory recall is common in case-control study designs because ‘cases’ may remember information about determinants more vividly and differently than ‘controls’. In addition, many farmers do not keep proper documentations and record of animals in their farms, a problem that is pervasive in extensive farms in developing countries. Temporality between cause and effect can also be confusing and difficult to ascertain because of the inherent limitations in case-control study design.

## Conclusion

The results of the present study revealed that the production system, presence of wildlife, presence of other non-cattle species on the same farm and history of abortion were important and significant risk factors associated with bovine brucellosis in Peninsular Malaysia. We believe that among these risk factors, the modifiable factor where changes can be implemented readily and with minimal financial implications would be to improve the biosecurity of farms by placing enclosures in the area through effective fencing. The presence of enclosures will reduce mingling between animals from separate herds and deter wildlife away from animal feed and water sources. We also suggest separating other species of animals from cattle herds to prevent infection from other non-cattle ungulates that may host the organism or result in cross-species *Brucella* infection. Educational program directed at farmers and collaboration between veterinary authorities and herd owners could further improve the management system. The lack of knowledge among farmers on the zoonotic nature of the disease is of concern and veterinary and human medical authorities need to improve public health education among high-risk populations in order to enhance precautionary measures and prevent disease occurrence. Finally, this study provides baseline information for further research on the bovine brucellosis and may be used to modify the level of disease present among herds in Malaysia.
